# Predicting medical usage rate at mass gathering events in Belgium: development and validation of a nonlinear multivariable regression model

**DOI:** 10.1186/s12889-022-12580-8

**Published:** 2022-01-25

**Authors:** Hans Scheers, Hans Van Remoortel, Karen Lauwers, Johan Gillebeert, Stijn Stroobants, Pascal Vranckx, Emmy De Buck, Philippe Vandekerckhove

**Affiliations:** 1 Centre for Evidence-Based Practice, Belgian Red Cross, Mechelen, Belgium; 2 Department of Public Health and Primary Care, Leuven Institute for Healthcare Policy, KU Leuven, Leuven, Belgium; 3 Humanitarian Services, Belgian Red Cross, Mechelen, Belgium; 4 Belgian Red Cross, Mechelen, Belgium; 5 Emergency Department, ZNA Stuivenberg, Antwerp, Belgium; 6 Department of Cardiology and Intensive Care, Jessa Ziekenhuis, Hasselt, Belgium; 7 Faculty of Medicine and Life Sciences, Hasselt University, Hasselt, Belgium; 8 Cochrane First Aid, Mechelen, Belgium; 9 Centre for Evidence-Based Health Care, Stellenbosch University, Cape Town, South Africa

**Keywords:** Mass gathering, Prediction model, Regression tree, Nonlinear regression model, Preventive medicine, Medical usage, Patient presentation rate, Transfer to hospital rate

## Abstract

**Background:**

Every year, volunteers of the Belgian Red Cross provide onsite medical care at more than 8000 mass gathering events and other manifestations. Today standardized planning tools for optimal preventive medical resource use during these events are lacking. This study aimed to develop and validate a prediction model of patient presentation rate (PPR) and transfer to hospital rate (TTHR) at mass gatherings in Belgium.

**Methods:**

More than 200,000 medical interventions from 2006 to 2018 were pooled in a database. We used a subset of 28 different mass gatherings (194 unique events) to develop a nonlinear prediction model. Using regression trees, we identified potential predictors for PPR and TTHR at these mass gatherings. The additional effect of ambient temperature was studied by linear regression analysis. Finally, we validated the prediction models using two other subsets of the database.

**Results:**

The regression tree for PPR consisted of 7 splits, with mass gathering category as the most important predictor variable. Other predictor variables were attendance, number of days, and age class. Ambient temperature was positively associated with PPR at outdoor events in summer. Calibration of the model revealed an *R*^2^ of 0.68 (95% confidence interval 0.60–0.75). For TTHR, the most determining predictor variables were mass gathering category and predicted PPR (*R*^2^ = 0.48). External validation indicated limited predictive value for other events (*R*^2^ = 0.02 for PPR; *R*^2^ = 0.03 for TTHR).

**Conclusions:**

Our nonlinear model performed well in predicting PPR at the events used to build the model on, but had poor predictive value for other mass gatherings. The mass gathering categories “outdoor music” and “sports event” warrant further splitting in subcategories, and variables such as attendance, temperature and resource deployment need to be better recorded in the future to optimize prediction of medical usage rates, and hence, of resources needed for onsite emergency medical care.

**Supplementary Information:**

The online version contains supplementary material available at 10.1186/s12889-022-12580-8.

## Background

Mass gatherings (MG) are defined by the World Health Organization (WHO) as “planned or spontaneous events where the number of people attending could strain the planning and response resources of the community or country hosting the event” [1].

To avoid MGs overloading the regular health care system, well-prepared and adequately equipped on-site health services are essential. For optimal use of resources (personnel, equipment, and finances) it is important to be able to predict patient load and health care needs. In the past 20 years, researchers have developed models to predict medical usage rates (MUR) at MGs, usually expressed as patient presentation rate (PPR) and transfer to hospital rate (TTHR) (e.g. [2–6]), and have validated these models by applying them to other MGs [7–9]. However, our recent systematic review of 16 prediction model development studies and three validation studies concluded that predictive performance and generalizability of these models is poor and we recommended the development of context-specific models [10].

Every year, volunteers of the Belgian Red Cross (BRC) provide onsite medical care at more than 8000 manifestations of all sorts, from local fairs and festivals, over recreational sports events, music concerts and dance events to large scale summer music festivals. An important fraction of these manifestations can be categorized as MGs. BRC’s Medical Triage and Registration Informatics System (MedTRIS) contains data on more than 200,000 medical interventions at MGs from the years 2006 to 2018, ranging in size from 2000 to more than 1 million attendees [11]. As such, it provides an excellent source of valuable data for the development and validation of a prediction model for MUR at MGs.

In this study, we aimed to develop multivariable prediction models of patient presentation rate (PPR) and transfer to hospital rate (TTHR) at MGs in Belgium, based on the MedTRIS database. Additionally, we wanted to validate these models and assess their generalizability, using more recent editions of the MGs used for model development (temporal validation) and an independent dataset of other MGs (external validation) [12].

## Methods

In the description of our methodology below, we adhered to the Transparent Reporting of a multivariable prediction model for Individual Prognosis Or Diagnosis (TRIPOD) statement for reporting of a prediction model study [13] (see Additional file 1).

### Study design and population

We applied a retrospective design to develop a prediction model for MUR at MGs in Belgium, making use of the MedTRIS database. MedTRIS is a web-based client server system for the registration of patients being treated at emergency medical services (EMS) by BRC at MGs in Belgium [11]. For each patient presentation at a care post, a patient encounter form (PEF) is completed by a BRC volunteer. The PEF contains such data as date and time of arrival, personal characteristics (age and sex), medical details (type of injury, triage category), and dismissal data (time, destination, means of transportation). Data collected on hard copy are immediately digitalized and saved in the MedTRIS database (using Access 2010; Microsoft Corporation). MedTRIS was implemented in its final form in 2006 and, as of 2018, contained medical data on more than 230,000 patient encounters at 411 MGs.

We pooled individual patient data stored in MedTRIS by edition of a MG, making MG (name and year of each manifestation) the unit of analysis. Year was not used to include a time component in the analysis of the dataset, but only to distinguish different editions of the same MG.

### Eligibility criteria

For the development of the prediction model, we included recurring MGs with: 1) at least five editions in the period 2009–2016; and 2) at least 10,000 attendees (cumulative for multiday events) at each of the editions included.

For the validation of the model, we created two separate subsets of the MedTRIS database. The first dataset contained data of the 2018 edition of those events that were included in the model development dataset. This type of validation is referred to as temporal validation [12]. The second validation dataset was the real external validation dataset, containing events from the period 2009–2016 that did not fulfil the inclusion criteria for the model development dataset. Thus, this heterogeneous validation dataset consisted of smaller MGs (2000 to 10,000 attendees), one-time MGs, and other MGs with less than five editions in the period 2009–2016.

### Outcomes

For each of the MGs included, we estimated PPR and TTHR as the outcomes for our prediction model. A review paper, calling for uniformity, advised to report PPR and TTHR per 1000 attendees [14], but because of relatively low numbers of patient presentations and transportations in our dataset, we decided to calculate PPR as the number of patients per 10,000 attendees and TTHR as the number of hospital transfers per 10,000 attendees to increase readability of these measures.

For PPR, all patient registrations at the EMS were included, irrespective of personal and medical characteristics or missing data for any of these characteristics. However, patient encounters registered the day before or after an official event day (typical for some of the bigger multi-day festivals with camping facilities), were excluded. For TTHR, the number of hospital transfers was calculated based on dismissal data on the PEF. Only transportations by ambulance were included; transportations by own means were excluded [14].

### Candidate predictors

Based on the conclusions from our systematic review on prediction modelling studies for MUR in MGs [10] and on the availability of data, we identified 11 candidate predictor variables for PPR: MG category, age of patients, time, number of days, number of attendees, camping, alcohol, indoor/outdoor, bounded/unbounded, ambient temperature, and humidity; and 3 additional candidate predictor variables for TTHR: distance and time to the nearest hospital, and predicted PPR. See Additional file 2 for more details on the predictor variables.

We retrospectively contacted organisers of each manifestation to obtain the number of attendees or participants. When a second request was left unanswered, we searched through online press archives to complete the list of attendances per event. For most multi-day events, only a cumulative number of participants was available, and for recreational sports events, the number of active participants (not including spectators) was used. Other predictor variables, such as the number of days, timing of the event, and presence of camping facilities were also collected from online press archives.

### Blinding

Assessment and, when applicable, categorisation (see Additional file 2) of predictor variables were done before (and hence, blinded to) calculation of PPR and TTHR. Because all patient registrations at EMS were used (regardless of time, triage category, or any other possible predictor variable), calculation of PPR was independent of any knowledge of the predictor variables. We matched the numbers of patient registrations or hospital transfers with the numbers of attendees to calculate PPR and TTHR only after having obtained all numbers of attendees.

### Handling of missing data

Even after two rounds of e-mails to organisers and a thorough search in online press archives, we missed some values for the number of attendees. For three outdoor music festivals, each lacking the number of visitors for 1 year in a series of at least 5 subsequent editions, we estimated the number for that year as the average of the previous and following year. For two indoor electronic dance music (EDM) events, only the first edition reported the number of attendees, mentioning that the event was sold out. Because all subsequent editions of both events were sold out and were held in the same venue, we copied the number of visitors from the first edition to the other editions.

Age was not completed in about 23% of all PEFs and referral place after dismissal from the care post was lacking in about 10% of all PEFs. We made no attempt to impute missing data. Hence, estimation of median age and TTHR for each event was based on approximately 77 and 90% of patient encounters, respectively.

### Regression tree analysis

#### PPR model

Our previous systematic review [10] concluded that classic linear or Poisson regression models poorly predicted MUR. Moreover, we also found poor predictive power when applying a preliminary multivariable Poisson regression model to our dataset. Therefore, and because many of our predictor variables were categorical or could easily be categorised, we developed a nonparametric prediction model making use of regression trees, with MG edition as the unit of analysis.

Within the domain of classification and regression trees (CART), regression trees are suitable for continuous outcome variables such as PPR and TTHR, and dichotomous or categorical predictor variables [15, 16]. A regression tree is built by splitting a parent node, into two child nodes (or leaves) that are as homogeneous as possible for the outcome variable, with the split determined by the most discriminating predictor variable. Next, child nodes are treated as parent nodes and split into new child nodes, again based on the predictor variable that maximizes the homogeneity of the resulting groups. This is continued until at a certain number of terminal nodes an equilibrium is reached between discriminatory power (fit of the dataset at hand) and robustness (predictive value for other datasets).

We entered three categorical (MG category, timing of event, availability of alcohol) and three dichotomous variables (camping, indoor vs outdoor, bounded vs unbounded) in their original form in the model. We rearranged number of days into three categories, and categorised number of attendees and age of the audience a priori. We created attendance classes aiming for approximately equal numbers in each class, but also taking into account gaps when arranging them from lowest to highest attendance. We created four age classes, mainly based on the target audiences of the different music and EDM events and subsequently defined by quantitative criteria. Thus, nine dichotomous or categorical variables were entered in the regression tree for PPR (see Additional file 2). In a first step, we performed univariable analyses by running separate models for each predictor variable. Then, all nine variables were entered in a multivariable regression tree, irrespective of their univariable performance.

We used the R package rcart to develop regression trees [17, 18]. Each regression tree was internally validated by 10-fold cross-validation. We used a complexity parameter (cp) of 0.01, which means that a new split must decrease the overall lack of fit by a factor of at least 0.01 before being attempted. We further tuned the model by combining all minsplit values (the minimum number of observations that must exist in a node in order for a split to be attempted) from 5 to 20 and all maxdepth values (the maximum depth of any node of the final tree) from 8 to 15 to identify the optimal hyerparameter setting. From these 128 resulting models, we selected the tree with minimal cross-validated error (xerror) (called pruning in the jargon of CART) to avoid overfitting [16]. The contribution of each predictor variable was not only assessed by counting the number of nodes for which it was the primary splitter, but also by calculating variable importance (VI). VI is a score for a variable’s ability to perform in a specific tree, either as a primary splitter or as a surrogate splitter (i.e. a variable whose pattern within the data set, relative to the outcome variable, is similar to the primary splitter at each node) [16]. As a measure of calibration or predictive performance, we calculated the *R*^2^ value for each regression tree as the square of the correlation coefficient between observed and predicted values. This value equals 1 – rel error from the R output.

#### Role of temperature

Because we hypothesized that the relationship between ambient temperature and PPR would be linear in the range of temperatures usually experienced during late spring and summer in Belgium [2, 10, 19–22], with no reasonable cut-off points for dichotomisation or categorisation, we did not enter temperature into the regression tree, but used it in a second phase to finetune the model.

We fitted a linear regression model for both the daily 24 h average (T_av_) and the daily maximum (T_max_) with PPR, making use of data from the 4-day outdoor music festival Rock Werchter (the only multi-day event with daily number of spectators and, hence, daily PPR, available). With 9 editions and 4 days per edition, we had 36 datapoints to assess the association between temperature and PPR. Based on T_max_ data for Rock Werchter, predicted PPR was adjusted to

$${PPR}_{T_{max}}={PPR}_{RT_i}+\frac{PPR_{RT_i}}{PPR_{RT RW}}\ast {b}_{T_{max}}\ast \left({T}_{max}-{T}_{{\mathit{\max}}_{RW}}\right)$$

Here, PPR_Tmax_ is the PPR adjusted for T_max_,; PPR_RTi_ the PPR estimated by the regression tree for event i; PPR_RTRW_ the PPR estimated by the regression tree for Rock Werchter; b_Tmax_ the slope; and T_maxRW_ the T_max_ for which PPR_Tmax_ = PPR_RTRW_. An analogous equation was used to estimate PPR_Tav_. See Additional file 3 for the elaboration of the equation.

The correction for temperature was performed for outdoor events organised from June to September only. All data for T_av_ and T_max_ were extracted from archives of MeteoBelgië [23], which in turn obtained its data from the Royal Meteorological Institute of Belgium (RMI). We compared *R*^2^ of the temperature-adjusted model with *R*^2^ of the crude regression tree to assess the added value of the temperature finetuning.

#### TTHR model

For the prediction of TTHR, we built a regression tree similar to the one for PPR, with the same nine predictor variables and identical classes for the categorical variables. We added time and distance to the nearest hospital, and we also included PPR as predicted with the unadjusted regression tree. We opted to include predicted PPR instead of observed PPR to better reflect the reality of predicting TTHR for an upcoming event. The modifying role of temperature was investigated analogously with the analysis for PPR.

#### Model validation

Predictor and outcome variables for the temporal and external validation datasets were assessed identically to those in the model development database. We evaluated the performance of the crude regression tree and the T_av_- and T_max_-adjusted models for PPR and TTHR by calculating the *R*^2^ values with corresponding 95% confidence intervals (CI).

## Results

### Descriptive results

#### Model development dataset

Two hundred two editions of 29 MGs met the inclusion criteria. After imputation of some attendance figures (see Methods), there were no missing data. We decided to exclude all 8 editions of the Dodentocht (a 100 km annual hiking event) because criteria for creating a PEF as well as definitions of triage categories on the PEF were very different compared to other MGs. Thus, 194 editions of 28 different MGs were included for the development of the prediction model. Detailed characteristics of the 28 included MGs are presented in Additional file 4.

Among these were 12 outdoor music events, 5 city festivals, 3 sports events, 3 outdoor EDM events, 3 indoor EDM events, and 2 indoor dance events. All sports events were active recreational events; no spectator sports events were included. The bulk of MGs were outdoor and bounded (127 editions of 18 different events), 36 MGs were outdoor and unbounded (5 different events), and 31 were indoor (5 different events). For most events, attendance class and age class were consistent across all editions, but e.g. DayDream grew from 12,000 visitors in 2010 (class 1) to 37,000 visitors in 2016 (class 3) and the age distribution of patients seen at Maanrock alternated between classes 2 (young adults) and 4 (mixed) in consecutive years.

An estimated total of 22,251,084 people attended the MGs used for model development. 131,181 patients were registered at EMS (average PPR 59/10,000 visitors), out of whom 2748 were transported to hospital by an ambulance (average TTHR 1.2/10,000 visitors). PPR and TTHR varied greatly among manifestations. The lowest PPR was recorded at Maanrock 2012 (a city festival with > 100,000 visitors, PPR = 3.9/10,000) and the highest at Dranouter 2009 (a folk music festival with approx. 25,000 visitors, PPR = 626/10,000). Five MGs (among which two editions of Blues Peer, a blues music festival with 20,000–25,000 visitors) had no hospital transportations whatsoever, whereas most editions of I Love Techno (indoor EDM, 35,000 visitors) had a TTHR of approximately 12/10,000.

#### Model validation datasets

Out of 28 MGs from the model development dataset (2009–2016), only 19 were also registered in MedTRIS in 2018. Especially, indoor EDM and outdoor EDM events were underrepresented in this temporal validation dataset, compared to the model development dataset. We excluded 18 out of 87 candidate MGs from the external validation dataset because of missing data for attendance (*N* = 11) or temperature (*N* = 4) or missing information on the event altogether (*N* = 3).

Thus, the temporal and external validation dataset contained 19 and 69 MGs, respectively. Their number of attendees was much lower (2,569,150 and 2,295,448 visitors, respectively) and, whereas their TTHRs were very similar to that of the model development dataset, overall PPR was notably lower for the external validation set (44/10,000 visitors).

Main characteristics of the model development dataset and validation datasets are summarized in Table 1.

**Table 1:** Characteristics of the datasets used for development and validation of the prediction model

	Model development^**a**^	Temporal validation^**b**^	External validation^**c**^
**Total nr. of manifestations (nr. of different manifestations)**	194 (28)	19 (19)	69 (32)
**Cumulative nr. of attendees (range)**	22,251,084 (10,000–1,500,000)	2,569,150 (5500–1,300,000)	2,295,448 (733–120,000)
**Cumulative nr. of patients (range)**	131,181 (24–7143)	17,084 (55–8229)	10,517 (13–716)
**Overall PPR (range)**	59.0 (3.9–626.3)	66.5 (4.4–290.9)	45.8 (2.7–411.8)
**Cumulative nr. of hospital transfers (range)**	2748 (0–113)	335 (0–98)	260 (0–15)
**Overall TTHR (range)**	1.2 (0.0–12.3)	1.3 (0.0–16.4)	1.1 (0.0–27.3)
**MG category, n (%)**			
**City festival**	37 (19)	3 (16)	10 (14)
**Indoor EDM**	19 (10)	1 (5)	0 (0)
**Indoor dance**	12 (6)	2 (11)	3 (4)
**Outdoor EDM**	21 (11)	1 (5)	18 (26)
**Outdoor music**	85 (44)	9 (47)	25 (36)
**Sports event**	20 (10)	3 (16)	13 (19)
**Attendance class, n (%)**			
**< 10,000**	0 (0)	0 (0)	13 (19)
**10,000–20,000**	39 (20)	4 (21)	20 (29)
**20,000–30,000**	36 (19)	5 (26)	10 (14)
**30,000–100,000**	67 (34)	4 (21)	21 (30)
**100,000–1,000,000**	44 (23)	5 (26)	5 (7)
**> 1,000,000**	8 (4)	1 (5)	0 (0)
**Age class, n (%)**^**d**^			
**Children (< 16 y)**	8 (4)	1 (5)	0 (0)
**Young adults (16–30 y)**	132 (68)	12 (63)	44 (64)
**Middle adults (> 30 y)**	32 (16)	3 (16)	16 (23)
**Mixed/family (broad range)**	22 (11)	3 (16)	9 (13)
**Timing, n (%)**			
**Day**	28 (14)	4 (21)	16 (23)
**Night**	51 (26)	5 (26)	9 (13)
**Day + night**	115 (59)	10 (53)	44 (64)
**Nr. of days (%)**			
**1**	80 (41)	8 (42)	33 (48)
**2**	39 (20)	2 (11)	21 (30)
**3+**	75 (39)	9 (47)	15 (22)
**Indoor, n (%)**	31 (16)	3 (16)	3 (4)
**Bounded, n (%)**	158 (81)	14 (74)	56 (81)
**Camping, n (%)**	78 (40)	8 (42)	26 (38)
**Availability of alcohol, n (%)**			
**None**	8 (4)	1 (5)	0 (0)
**Limited**	20 (10)	3 (16)	13 (19)
**Unlimited**	166 (86)	15 (79)	56 (81)

*PPR* patient presentation rate, *TTHR* transfer to hospital rate, *MG* mass gathering, *EDM* electronic dance music

^a^Dataset containing the 2009–2016 editions of MGs with at least five editions in the period 2009–2016 and at least 10,000 attendees (cumulative for multiday events) at each of the editions included

^b^Dataset with the 2018 editions of MGs included in the model development dataset

^c^Dataset with the 2009–2016 editions of MGs not meeting the inclusion criteria for the model development dataset

^d^Median values were used. For the mixed/family class, a combination of Q1 < 21 y with IQR > 20 y was applied. When Q1 < 18 y, an IQR > 15 y was deemed sufficient to fit in the mixed/family class

### Prediction of PPR

#### Univariable regression tree

Each univariable regression tree was built on data for all 194 MGs. Additional file 5 provides an overview of the results for each predictor variable. In brief, MG category had the highest prognostic value (*R*^2^ = 0.36), with the lowest PPR predicted for city festivals (PPR = 2/10,000) and the highest for outdoor EDM and outdoor music events (PPR = 148/10,000). Camping had a relatively high *R*^2^ of 0.22, with lower PPR for no camping (PPR = 67/10,000) than for camping (155/10,000). *R*^2^ values of timing, number of days, indoor vs outdoor, and alcohol were too low for a relevant univariable association with PPR.

#### Multivariable regression tree

With 194 outcome events and 9 and 12 candidate predictor variables for PPR and TTHR, respectively, we had 21.6 events per variable (EPV) for PPR and 17.6 EPV for TTHR, both well above the advised number of 10 EPV [1].

**Fig. 1:**
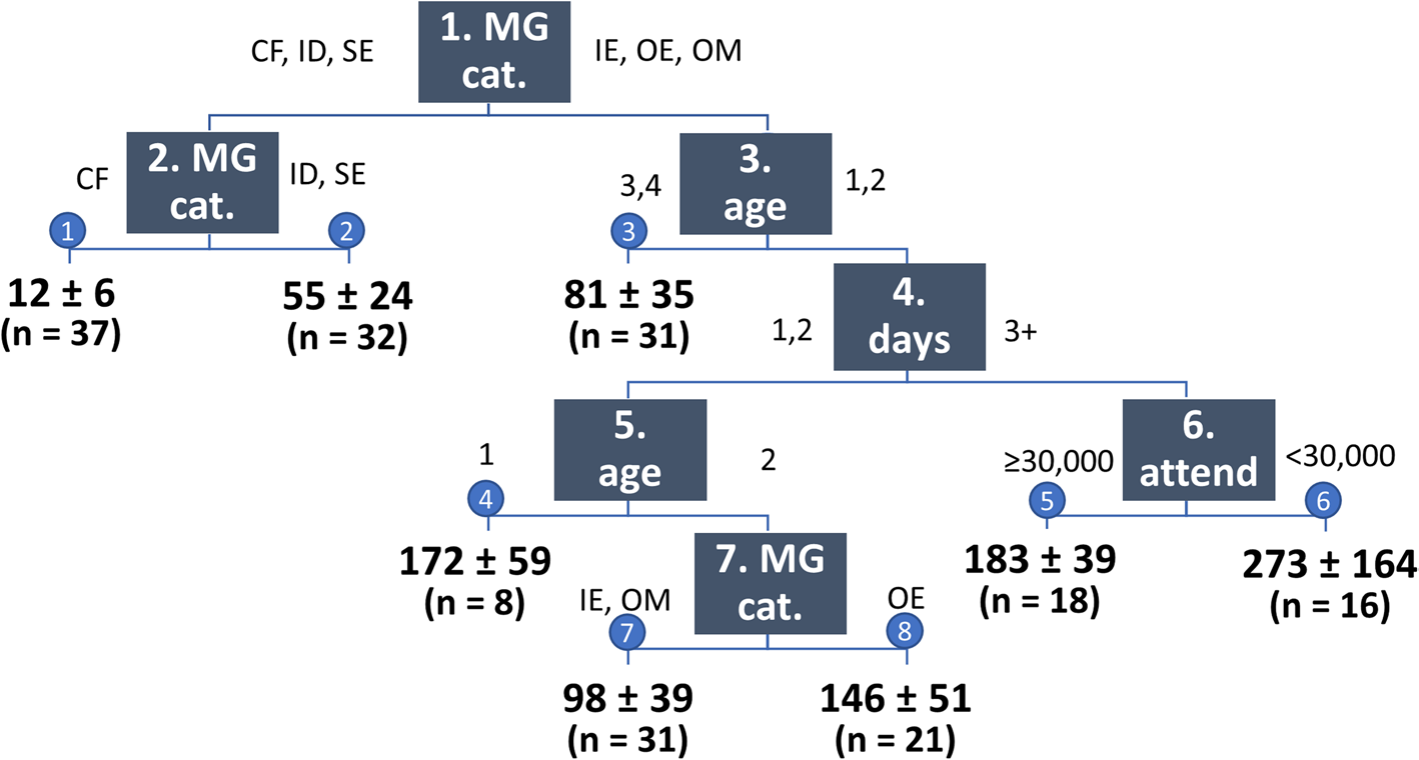
Multivariable regression tree for PPR. Each square contains the hierarchical number of the split and the name of the splitting variable (MG cat.: MG category; age: age class; days: number of days; attend: attendance). CF: city festival; IE: indoor EDM; ID: indoor dance; OE: outdoor EDM; OM: outdoor music; SE: sports event. See Table 1 for age classes. Each blue circle contains the number of the terminal node. Numbers in bold indicate the predicted PPR per 10,000 visitors for each terminal node (mean ± standard deviation) and the number of MGs (n) in this node

When all predictor variables were entered in the model for PPR, the most parsimonious regression tree consisted of 7 splits and, hence, 8 terminal nodes. In Fig. 1, each terminal node shows the predicted PPR for the MGs in that node, estimated as the average value with standard deviation of the observed PPRs of the included MGs. As expected from the univariable analyses, MG category was the most important predictor variable, determining 3 splits, among which the first 2. These splits were identical to those in the univariable analysis with MG category. Mathematically, MG category had a VI of 28%. Other predictor variables in the regression tree were attendance class (1 split, VI 16%), number of days (1 split, VI 11%), and age class (2 splits, VI 7%). Although never selected as best splitting variable, bounded vs unbounded and camping vs no camping had a higher VI than age class (12 and 11%, respectively), because they acted as good surrogate variables at several splits.

#### Temperature-adjusted predictions

We found a strong linear association of both T_av_ and T_max_ with PPR on the Rock Werchter festival. For T_av_, the linear regression equation was:

$${PPR}_{T_{av}}={PPR}_{RT_i}+\frac{PPR_{RT_i}}{183}\ast 10.6\ast \left({T}_{av}-20.4\right)$$

for any MG i that has a PPR predicted by the regression tree (PPR_RTi_). Analogously, for T_max_, the regression equation was:

$${PPR}_{T_{max}}={PPR}_{RT_i}+\frac{PPR_{RT_i}}{183}\ast 7.6\ast \left({T}_{max}-25.6\right)$$

for any MG i. In doing so, we adjusted the PPR_RTi_ for outdoor events from June to September to PPR_Tav_ or PPR_Tmax_. See Additional file 3 for the intermediate steps.

#### Predictive performance

By adding more predictor variables to the model, *R*^2^ increased from 0.36 for the univariable association with MG category to 0.63 (95% CI 0.55–0.71) for the multivariable regression tree. By applying the temperature equations to outdoor MGs held in the period from June to September, the *R*^2^ of the temperature-adjusted regression tree slightly increased further to 0.67 (95% CI 0.59–0.74) for T_av_ and 0.68 (95% CI 0.60–0.75) for T_max_.

**Fig. 2:**
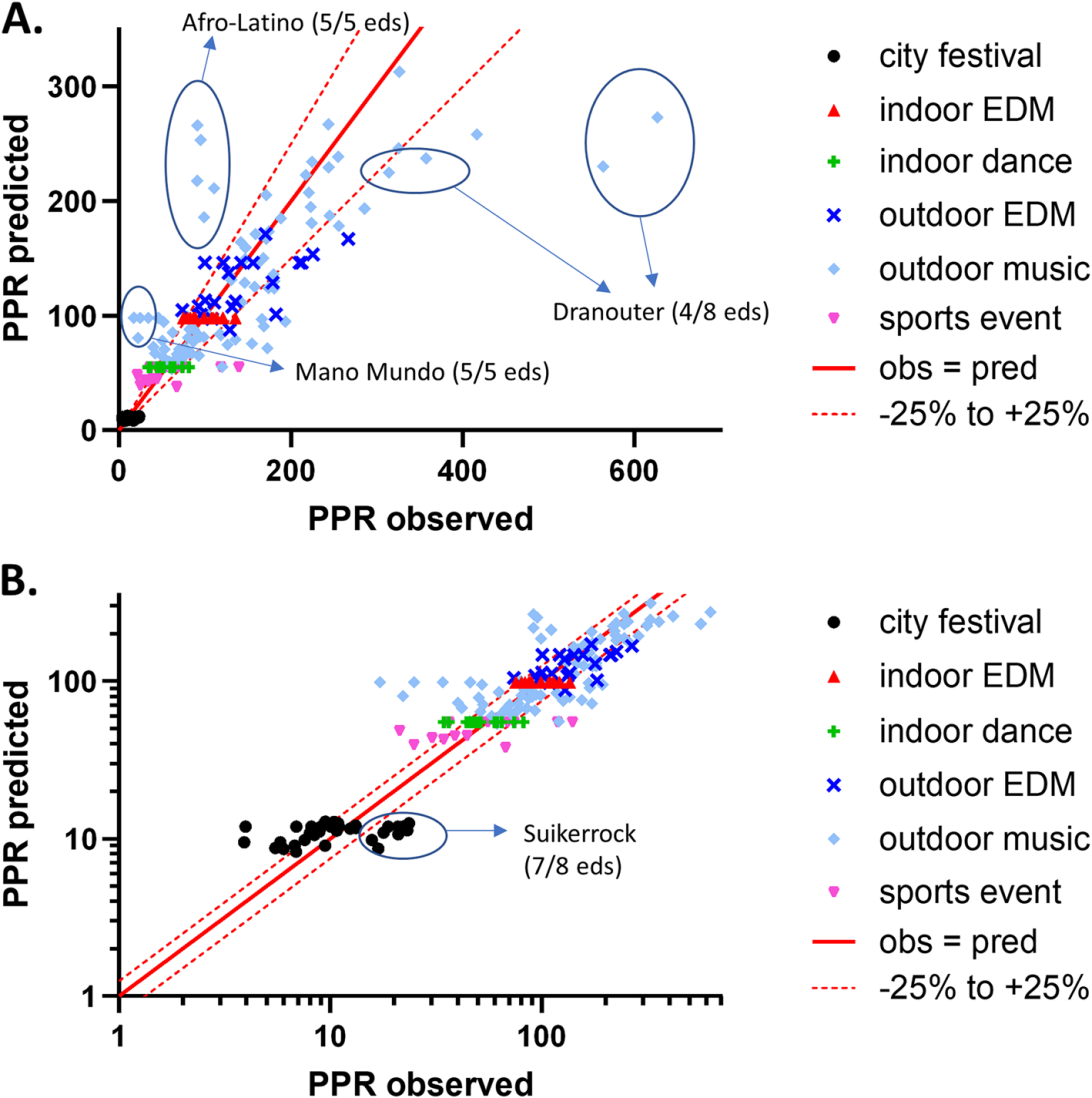
Calibration of the PPR regression tree adjusted for T_max_. **a** Calibration for the dataset used for model development on a linear scale; **b** the same data plotted on a logarithmic scale. The solid red line represents a perfect match between the observed and predicted PPR; the dotted red lines indicate 25% under- or overestimation

Figure 2 gives a visual presentation of the calibration of the temperature-adjusted model on a linear scale (Fig. 2A) and on a logarithmic scale (Fig. 2B). For 107 out of 194 MGs, predicted PPR values were within a 25% deviation of the observed PPR. PPR of two outdoor music events, Afro-Latino and Mano Mundo (each having 5 editions in the dataset), was systematically overpredicted with more than 50% (and up to a factor of 5.7 for one edition of Mano Mundo). In contrast, PPR was higher than predicted for 4 editions of Dranouter, another outdoor music festival; for two editions, the observed PPR was more than double the predicted PPR. For 7 out of 8 editions of the city festival Suikerrock, the observed PPR was, although generally low, more than 50% higher than the predicted PPR.

### Prediction of TTHR

#### Univariable regression tree

Again, each univariable regression tree contained data for all 194 MGs. Results of 12 univariable analyses are presented in Additional file 5. MG category and predicted PPR had the highest prognostic value (*R*^2^ = 0.34 and *R*^2^ = 0.32, respectively). Distance and time to the nearest hospital were unrelated to predicted TTHR.

#### Multivariable regression tree

**Fig. 3:**
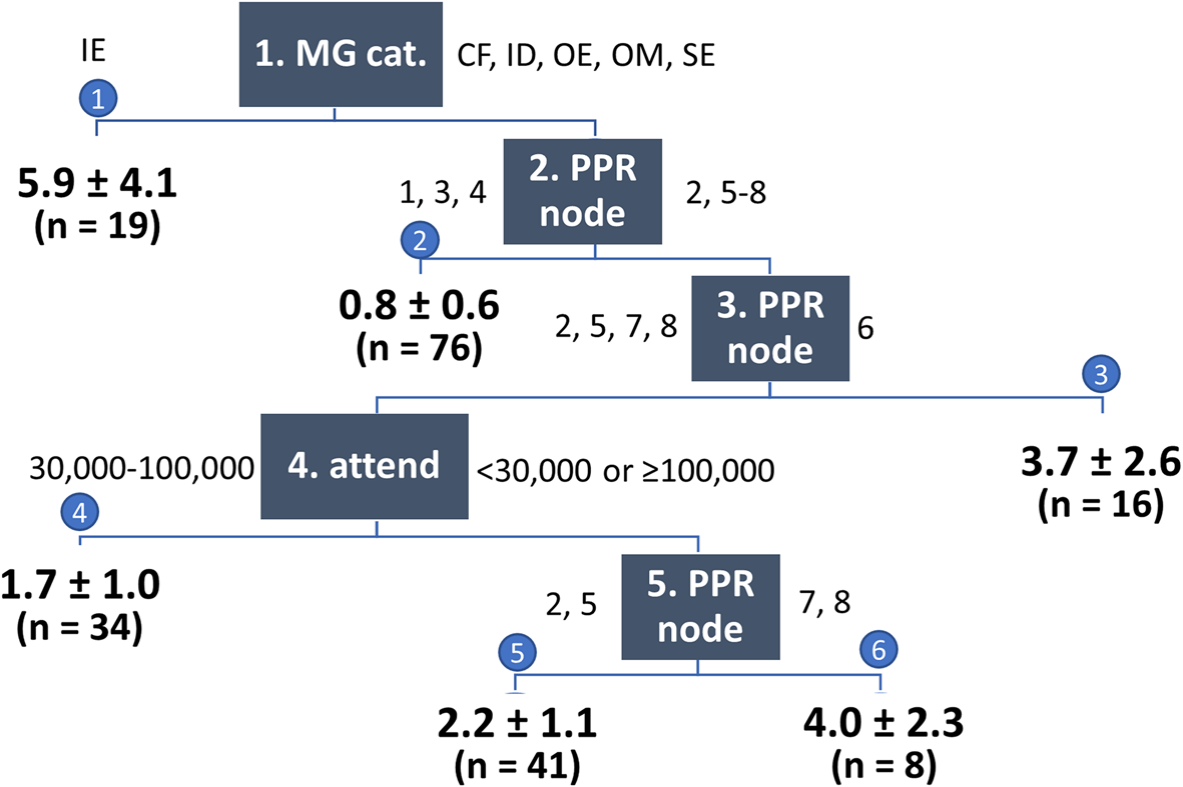
Multivariable regression tree for TTHR. Each square contains the hierarchical number of the split and the name of the splitting variable (MG cat.: MG category; node: number of terminal node in the PPR model, see Fig. 1; attend: attendance). CF: city festival; IE: indoor EDM; ID: indoor dance; OE: outdoor EDM; OM: outdoor music; SE: sports event. Each blue circle contains the number of the terminal node. Numbers in bold indicate the predicted TTHR per 10,000 visitors for each terminal node (mean ± standard deviation) and the number of MGs (n) in this node

For TTHR, the most parsimonious model with good performance was a regression tree with 5 splits, resulting in 6 terminal nodes (Fig. 3). The tree was almost entirely determined by predicted PPR and MG category. Predicted PPR determined 3 splits and had the second highest VI (31%). Although MG category determined only one split, it had the highest VI (41%), because this was the first split and MG category partly overlapped with predicted PPR. Attendance class was the discriminatory variable in the last remaining node and had a VI of 5%.

#### Temperature-adjusted predictions

In contrast to PPR, TTHR at Rock Werchter was not associated with T_av_ or T_max_. The respective regression equations were

$${TTHR}_{T_{av}}=1.3+0.3\ast {T}_{av}$$

with *N* = 36; *p* = 0.37; *R*^2^ = 0.02 and

$${TTHR}_{T_{max}}=1.7+0.0\ast {T}_{max}$$

with *N* = 36; *p* = 0.93; *R*^2^ = 0.00. Hence, TTHR values that were predicted with the regression tree were not adjusted for temperature.

#### Predictive performance

**Fig. 4:**
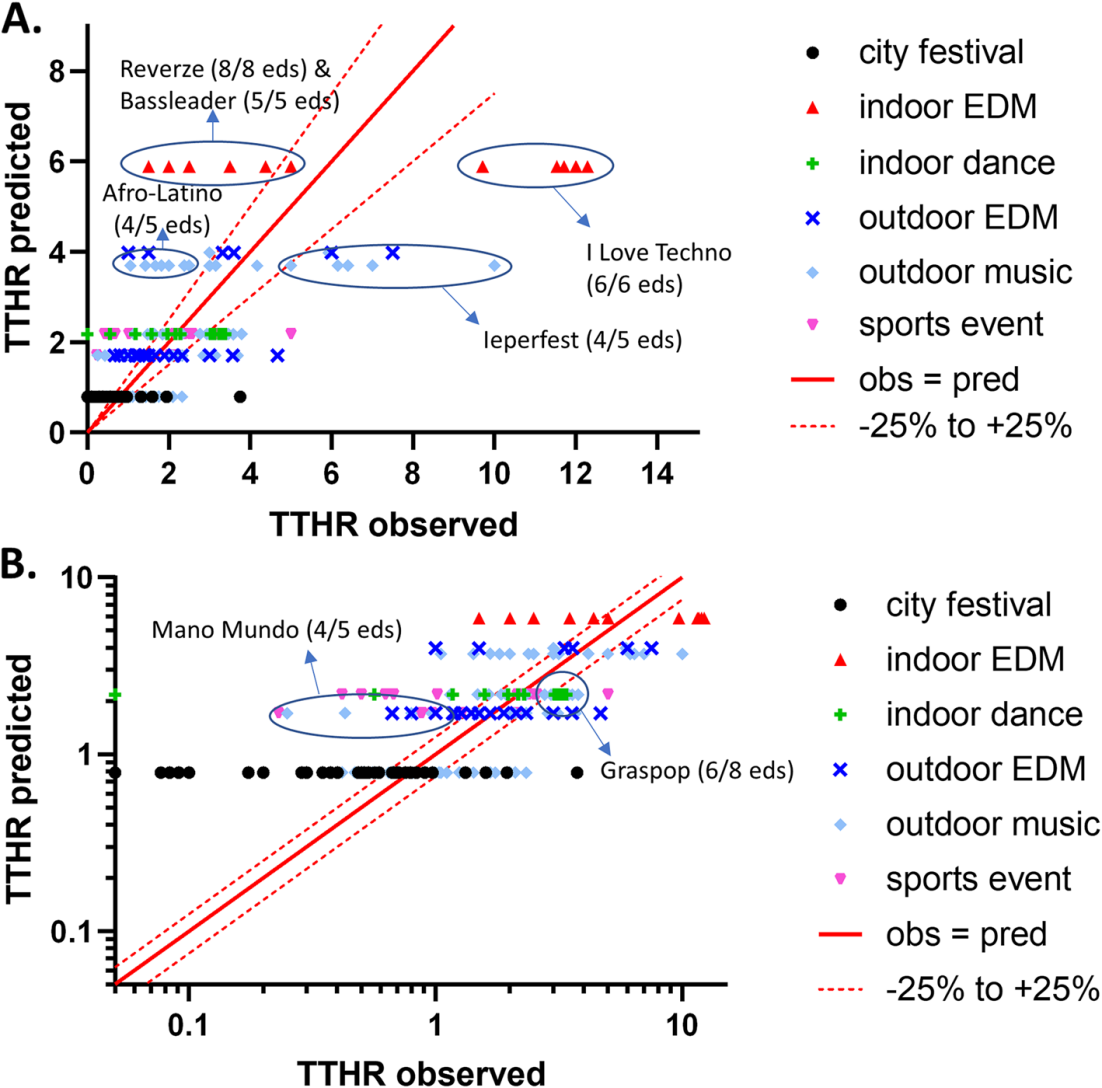
Calibration of the TTHR regression tree. **a** Calibration for the dataset used for model development on a linear scale; **b** the same data plotted on a logarithmic scale. The solid red line represents a perfect match between the observed and predicted TTHR; the dotted red lines indicate 25% under- or overestimation

The *R*^2^ of the multivariable regression tree for TTHR was 0.48 (95% CI 0.38–0.57). Figure 4 gives a visual presentation of the calibration of the model on a linear scale (Fig. 4A) and on a logarithmic scale (Fig. 4B). For 49 out of 194 MGs, predicted TTHR values were within a 25% deviation of the observed TTHR. As with PPR, TTHR at Afro-Latino and Mano Mundo (among others), was systematically overpredicted. Within the indoor EDM category, a TTHR of 5.9 was an overprediction for Bassleader and Reverze and an underprediction for I Love Techno. TTHR at outdoor heavy metal music festivals Graspop and Ieperfest was also underpredicted.

### Validation of PPR prediction model

#### Temporal model validation

**Fig. 5:**
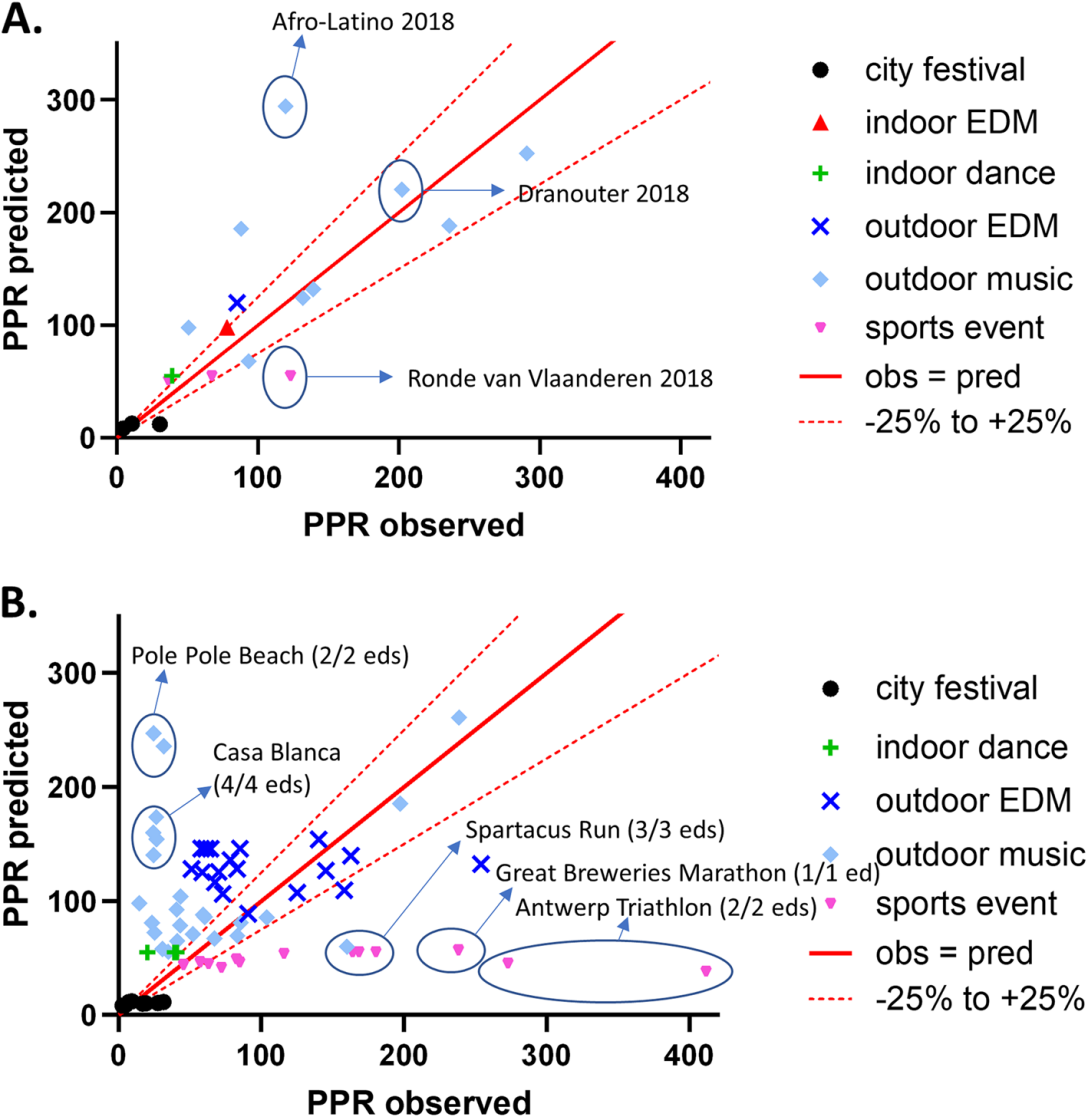
Validation of the multivariable PPR prediction model. **a** Temporal validation of regression tree, adjusted for T_max_, with the 2018 dataset. **b** External validation of regression tree, adjusted for T_max_, with other 2009–2016 manifestations. The solid red line represents a perfect match between the observed and predicted PPR; the dotted red lines indicate 25% under- or overestimation

Predicted PPR for the 19 manifestations in 2018 was estimated by first applying the multivariable regression tree to the characteristics of each manifestation, followed by an adjustment for T_av_ or T_max_, using the same equation as in the model development phase. Predictive value of the unadjusted model was fairly high with an *R*^2^ of 0.64 (95% CI 0.29–0.85), but in contrast to the data for model development, inserting temperature in the model slightly decreased *R*^2^ to 0.60 (95% CI 0.24–0.83) and 0.61 (95% CI 0.25–0.83) for T_av_ and T_max_, respectively. Figure 5a shows the temporal validation for the T_max_-adjusted prediction model. Again, PPR for Afro-Latino was underpredicted, but PPR at Dranouter 2018 was predicted very well by the model.

#### External model validation

Analogously, PPR was predicted for 69 manifestations that did not meet the criteria for inclusion in the development dataset. The model showed poor predictive value for this set of manifestations: *R*^2^ values were 0.03 (95% CI 0.00–0.15), 0.02 (95% CI 0.00–0.13) and 0.02 (95% CI 0.00–0.14) for the crude model, the T_av_-adjusted model and the T_max_-adjusted model, respectively. Especially PPRs of some intensive sports events (the Antwerp Triathlon, an Olympic distance triathlon for professional and recreational athletes; the Great Breweries Marathon; and the Spartacus Run, an obstacle running race) were highly underpredicted (Fig. 5b). On the other hand, PPRs at Pole Pole Beach (a world music festival, similar to Afro-Latino and Mano Mundo) and Casa Blanca (also an outdoor music event) were highly overestimated by the prediction model.

### Validation of TTHR prediction model

#### Temporal model validation

**Fig. 6:**
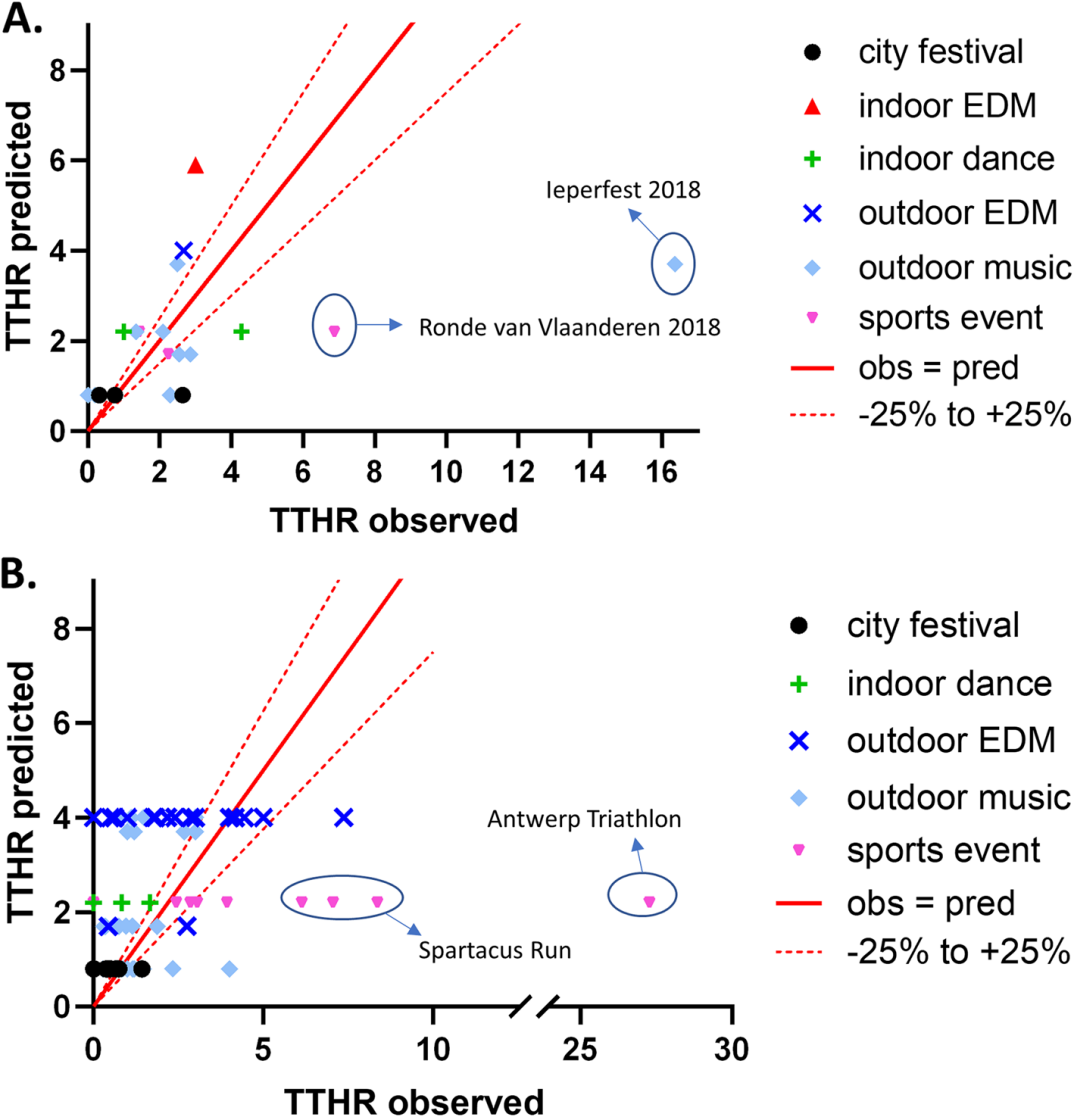
Validation of the multivariable TTHR prediction model. **a** Temporal validation of regression tree with the 2018 dataset. **b** External validation of regression tree with other 2009–2016 manifestations. The solid red line represents a perfect match between the observed and predicted TTHR; the dotted red lines indicate 25% under- or overestimation

*R*^2^ for the TTHR model, applied to the 2018 dataset, was 0.16 (95% CI 0.00–0.52), which is remarkably lower than that obtained for the development data set (*R*^2^ = 0.48). Especially, TTHR was highly underestimated for Ieperfest, as can be seen in Fig. 6a. As with PPR, TTHR was also underestimated for the Ronde van Vlaanderen (a recreational cycling event).

#### External model validation

For manifestations that were not included in the development dataset, *R*^2^ of the model for TTHR was 0.03 (95% CI 0.01–0.15), which was also much lower than the value for model development. TTHR for the Antwerp Triathlon (1 edition) and the Spartacus Run was highly underpredicted, whereas TTHR for most outdoor EDM events and all indoor dance events was overpredicted (Fig. 6b).

## Discussion

### Main predictors of PPR

The best tree for PPR at 194 editions of 28 different MGs in Belgium contained 8 terminal nodes, with predicted PPR ranging from 12/10,000 (all city festivals) up to 273/10,000 (3 outdoor music festivals).

#### Mass gathering category

MG category, having the highest VI (28%) and determining 3 splits among which the first 2, was clearly the most important predictor variable in the regression tree. This is in line with results of a recent study that used multivariable regression trees for prediction of PPR at a wide variety of more than 200 MGs in Australia [24]. In a recent systematic review [10], we found that MG category was significantly associated with PPR in 5 out of 6 studies that included MG category as a predictor variable for PPR [2, 5, 20, 24–26].

However, categorization of events is very study-specific, hampering a direct comparison between our results and those found in the literature. Indeed, some studies confirmed our finding that sports events had lower PPR than outdoor music concerts [2, 26], but those were spectator sports events in stadiums, in contrast to active amateur sports events in our current study. Similarly, two studies found differences in PPR between several music genres [5, 25], whereas we only differentiated between EDM and “other” music (including heavy metal, rock, folk, world music, …).

#### Ambient temperature

In our systematic review [10], we found that temperature (including heat index, a combined measure of air temperature and relative humidity) was positively associated with PPR in 5 out of 7 papers that evaluated ambient temperature at outdoor events [2, 5, 22, 24–27]. This relationship is confirmed by our current analysis.

We did not dichotomize temperature (which would be necessary to insert it in the regression tree) because the cut-off values reported in the literature for increased PPR [5, 26] are at the end of or beyond the usual range of summer temperatures in Belgium. We used the original continuous data instead to adjust the predicted PPR from the regression tree for outdoor events organised in summer. This slightly improved model performance Our approach is justified by the observation that we found a highly significant linear relationship between daily maximal temperature and PPR with all other potential predictor variables kept constant.

We did not use heat index, because the circumstances in which relative humidity substantially affects the apparent temperature (i.e. temperatures > 27 °C combined with a relative humidity > 60%) are not encountered in the maritime temperate climate of Belgium.

#### Other variables

Attendance did not seem to be an important variable in the model. With a VI of 16%, it appeared only once in our regression tree, and in a terminal split, which means that it only discriminated among otherwise similar MGs (outdoor music events of 3 days or more). Not surprisingly, attendance has been positively associated with the absolute number of patient presentations [2, 24], but PPR on music concerts, a relative measure, was not related to attendance in another study [25].

Age class had a VI of 7% and defined two splits in the part of the regression tree containing EDM and outdoor music events. PPR was lowest when older adults were present (age classes 3 and 4) and, among events of 1 or 2 days, it was highest at one manifestationthat was organized for an exclusively minor audience. To the best of our knowledge, age distribution has not been shown a predictor of PPR at EDM or music events before [10, 21].

Although this variable was not retained in the final model, we found clearly higher PPR for bounded than for unbounded events (see Additional file 5). Since contradicting evidence has appeared in the literature [2, 20], it is not clear whether this result reflects a real increase of risk for sickness or injury at bounded events or simply a higher visibility of care posts and less “leakage” of patients to other medical facilities [2].

We found higher PPR at outdoor music events consisting of 3 days or longer than at those of 1 or 2 days (VI 11%). As far as we know, number of days has not been described as a predictor variable for PPR. A couple of studies have described an increasing PPR during the course of a multi-day event [19, 28], which may explain our finding. However, we could not evaluate the progression of PPR during multi-day events because we failed to obtain attendance figures by day.

### Predictors of TTHR

The best regression tree for TTHR contained 6 terminal nodesPredicted TTHR was almost entirely determined by predicted PPR (3 nodes, VI 31%) and MG category (1 node, VI 41%). The highest TTHR was found for indoor EDM (5.9/10,000). For MG categories other than indoor EDM, the association between predicted PPR and TTHR was not entirely straightforward. In particular, sports events (predicted PPR of 55/10,000) and events with a predicted PPR as high as 183/10,000 had the same predicted TTHR (either 1.7/10,000 or 2.2/10,000).

In contrast to PPR, TTHR was clearly not affected by ambient temperature. Temperature-affected injury categories are mostly sunburn and headache, and these usually do not require referral to a hospital.

Our findings are in line with results found in other studies [2, 5, 22, 24], although the body of evidence for prediction of TTHR is much lower than that for PPR.

### Model performance

Internal validation, or calibration, of the regression trees revealed an acceptable predictive power, especially for the temperature-adjusted PPR model (*R*^2^ = 0.68). Two studies included in our systematic review [10] obtained similar *R*^2^ values for their multivariable prediction model [2, 4], whereas another one failed to explain variation in PPR (*R*^2^ = 0.04) [25].

Within smaller MG categories, such as city festivals, indoor dance and sports events, observed PPR was relatively homogeneous, and hence, predicted by one single value for the entire MG category. MGs with the highest under- or overprediction of PPR were some typical summer festivals, i.e. multi-day music festivals on a large meadow outside the town center with onsite camping facilities. More specifically, PPR of most editions of Dranouter were dramatically underestimated by the model, and PPR of all Afro-Latino editions was systematically overpredicted. Still, these festivals are not very different in music genre (mainly folk music and world music, respectively), duration (4 days and 3 days, respectively), and number and age distribution of spectators. Visibility and accessibility of EMS may partly explain the difference between observed and predicted PPR at Dranouter. In 2009 and 2010, there were two onsite care posts at Dranouter, and these were the years with the highest PPR (> 500/10,000). From 2011 on, there was only one care post, and PPR dropped below 350/10,000 from that year on, which is much more in line with the predicted PPR for this festival.

Characteristics of EMS are also important when interpreting the results for TTHR. The most striking feature of Fig. 4 is the discrepancy between TTHR at I Love Techno and TTHR at other indoor EDM events (Bassleader and Reverze). The care post at I Love Techno is more limited in space than that at the other two events (both held at the same venue, but different from that of I Love Techno), which is why there was an agreement with the nearest hospital to transfer relatively early to make room for new patients presenting.

In general, TTHR appears to be harder to predict than PPR, as indicated by our clearly lower *R*^2^ of 0.48 and by a very similar difference in performance between a prediction model for PPR and one for TTHR, obtained by Arbon et al. [2]. This is not surprising for several reasons. First, TTHR is partly estimated by predicted PPR, adding another level of uncertainty to the model. Second, TTHR is highly dependent on the qualification of personnel at the care posts. Finally, the absolute number of hospital transfers is low at most MGs, and small differences in absolute numbers are enlarged when presented in relative figures such as TTHR.

### Model validation

#### Validation of the PPR prediction model

Temporal validation of the temperature-adjusted PPR model revealed an acceptable *R*^2^ of 0.61. However, when externally validating the model with other manifestations from the MedTRIS database, *R*^2^ dropped to only 0.02, reflecting a very weak predictive power. PPR at the Antwerp Triathlon (2 editions), the Great Breweries Marathon (1 edition), and the Spartacus Run (3 editions) was highly underestimated. These are very demanding events characterized by either a limited and competitive field of participants (triathlon and marathon), or by a trail littered with several obstacles that increase the risk of injury (Spartacus Run). In contrast, the three different sports events that were used to develop the model were generally recreational in nature: both the Gordelfestival and Ronde van Vlaanderen are cycling tours with no fixed start time or time registration, and the Antwerp 10 miles, although a timed running race, has a large and predominantly recreative field of participants. Thus, further finetuning of the prediction model, by discriminating between types of sports events, is warranted.

On the other hand, PPR at outdoor music events Pole Pole Beach and Casa Blanca was highly overpredicted. Pole Pole Beach is a world music festival, much alike to Afro-Latino, whose PPR was also overpredicted by the model. Casa Blanca was a family-oriented festival, that has retired after 2013. As suggested by results obtained by others [5, 25], splitting outdoor music festivals into categories such as “world music”, “rock music”, and “heavy metal” (because of the high PPR and TTHR at Graspop and Ieperfest, two heavy metal festivals in the development dataset) would probably increase predictive power of the model.

#### Validation of the TTHR prediction model

*R*^2^ of the TTHR model dropped to 0.16 for temporal validation, and to 0.03 for external validation. Ieperfest (a heavy metal festival known for its mosh pit culture), the Antwerp Triathlon and Spartacus Run were the most prominent outliers in terms of underpredicted TTHR. Additionally, the model was clearly not sufficiently discriminative for outdoor EDM events, which all had a predicted TTHR of 4.0/10,000, but varied in reality from 0/10,000 to 7.4/10,000.

Although validation of the model showed poor performance for TTHR, over- or underprediction was marginal in absolute values. For example, the very high TTHR of 27/10,000 for the Antwerp Triathlon was caused by only two hospitalizations for 733 participants. The highest underprediction in terms of absolute hospitalizations per event day was found for I Love Techno, with around 20 hospitalizations more than predicted at each of 6 editions. However, as explained above, the working conditions for healthcare workers at this indoor EDM event are known and can be anticipated for by providing more ambulances and making good agreements with the nearest emergency department.

### Strengths and limitations

#### Pros and cons of regression trees

We analyzed our data with regressions trees, a statistical tool from the CART family. This method has some advantages compared to linear regression. First, the result of CART analysis, i.e. the regression tree, is very easy to implement and interpret. The predicted value for the outcome is found by simply following subsequent splits from top to bottom, based on the known values of the predictor variables. Second, being a non-parametric method, no assumptions need to be made about linearity of the association or distribution of the variables. Issues such as multicollinearity, complex interactions, and outliers are also dealt with by the specific design of CART. Finally, missing values can be replaced by surrogate variables [16, 29].

However, CART analysis presents some disadvantages as well. First, it is not very suitable for continuous predictor variables. When these are inserted in the model, they will be dichotomized automatically to create the best fitting model, a practice that is strongly discouraged [13]. Although still leading to loss of data, categorization of continuous data should be done before attempting to run the model. In our dataset, we had only three continuous variables: attendance, age and temperature. Attendance ranged from 10,000 to over 1000,000 and was split into five categories. We created four classes for the age distribution of patients, not only taking into account median age, but also the IQR, thus making use of as much information as possible. We were able to keep the temperature variable in its original continuous form, by not inserting it in the regression tree, but adjusting the results from the regression tree with a regression equation based on a linear association between ambient temperature and PPR instead. That way, we combined the advantages of regression trees with those of linear modelling.

A second drawback is that a regression tree is prone to instability: small changes in the data can result in completely different trees. Finally, as with linear models, overfitting is a risk in CART modelling as well [16, 29]. We applied 10-fold cross-validation to counteract the risks of instability and overfitting [12, 29]. Additionally, we limited the number of candidate predictor variables to those found relevant in the literature [10] to ensure that EPV would be higher then 10, which is a rule of thumb to prevent overfitting [13].

#### Assessment of predictor and outcome variables

Our regression tree indeed failed to predict PPR and TTHR for specific types of manifestations, and certainly for those that were different from the events the tree was built upon. This finding suggests that some important predictors of MUR have been overlooked, or at least not optimally used, in our model.

MG category was an important determinant of predicted PPR and TTHR, but there is room for improvement. As discussed above, outdoor music is a broad category, containing one-day concerts to four-day festivals, and music genres such as folk music, world music, rock, and heavy metal. Adding either a categorical variable with music genres [5, 25] or a variable representing “crowd mood”, which is assumed to be related to music genre [21], can probably improve predictive power of the model. The same is true for the category of sports events, which may be split into running vs cycling, and/or competitive vs recreational. However, the current number of sports events in the MedTRIS database is too small to allow for sufficiently stuffed subcategories.

Ambient temperature was not measured onsite. Instead, we retrospectively used data central data from the central weather station in Uccle (Brussels), operated by the RMI. Although Belgium is a small country with limited variation in elevation and latitude, daily maxima may be more than 10 °C higher in the Campine region than at the coast on warm spring and summer days.

Consumption of alcohol and other drugs is assumed [20, 21, 25] and has been shown [30] to increase patient needs. However, we have no data on the prevalence of drugs use at the MGs in MedTRIS, and we could only roughly estimate alcohol consumption by a variable indicating “no” (a festival for children only), “limited” (active sports events) or “unlimited” (all other events) access to alcohol.

Proper prediction of PPR and TTHR is important for organizations such as BRC to optimize onsite medical care at MGs, but in reverse, characteristics such as number, location, visibility, and size of care posts can also influence the influx of patients, as illustrated anecdotally with the examples of I Love Techno and Dranouter above. Unfortunately, information on these characteristics of medical care provided at MGs in MedTRIS was scattered and unusable for our analysis.

The number of attendees is indispensable to calculate PPR and TTHR. Yet, it was not recorded in the course of the years, and we had to construct this variable retrospectively by contacting organisers and sifting through online press releases. As a result, we obtained rough estimates (usually rounded to the nearest 1000) for most MGs and needed to estimate attendance for some events by interpolation or deduction (see Methods). Moreover, for multi-day MGs (except Rock Werchter), we obtained only the overall number of attendees. As a consequence, we treated these MGs as a single data line and used temperatures averaged over the course of the event, thus missing potential fluctuations in PPR due to weather conditions.

We had no missing data for other predictor or outcome variables, but referral place after dismissal from the care post was lacking in about 10% of all PEFs. Because the vast majority of patients qualified as “back to the event” (94% of all patient presentations), it is possible that this category was more easily forgotten when completing the PEF than the rare (2% of presentations) and more important category of “transported to hospital by ambulance”. This would imply that overall TTHR was slightly overestimated in our analysis.

#### Predictive performance

From the results and discussion above, it is clear that the prediction model performs well for PPR on future editions of most MGs included in the development dataset, but fails to predict PPR and TTHR at many other MGs. This limitation is not new, as earlier models for PPR and TTHR prediction [2, 3, 6] all revealed poor predictive performance and generalizability when applied to external datasets [7–10].

Just like earlier prediction models for MGs [2–6, 24], our model predicts overall PPR and TTHR, regardless of temporal variation during the event. However, PPR, and hence the need of medical care, fluctuates over the course of the event.

### Implications for practice and future research

As explained above, MG category should be refined into music genres and types of sports events for better model performance. Also, time of admission to the care post is a key variable to model temporal variation in PPR. Such an increase in variables and variable categories is only possible when the number of events per variable and events per category is sufficiently high to avoid overfitting.

Based on our observations, important event-specific variables need to be systematically recorded, such as the number of attendees (both the estimated number before the start and the eventual number for each day of the event), onsite temperature (also for indoor conditions, as the lack of ventilation or air conditioning may contribute to the occurrence of heat-related conditions), and a detailed description of deployment of resources (number, location and capacity of EMS, number and qualification of crew, presence of emergency doctors, number of ambulances, etc.).

BRC will use the results of this study to predict future MUR and hence, to provide sufficient personnel and materials at MGs. At the same time, new data will be systematically collected in a larger variety of MGs for further elaboration of the prediction model. However, during most of the years 2020 and 2021, all MGs in Belgium have been cancelled due to the COVID-19 crisis. Recently, the Belgian Federal Public Health Service developed a separate prediction model for patient encounters at music MGs in Belgium [31, 32]. The compatibility of both models towards a better prediction of MUR needs to be explored.

In normal years, BRC provides onsite medical care at more than 8000 manifestations, many of which do not classify as MGs. These smaller manifestations were not used for model development or validation because patient encounters have not been systematically recorded in MedTRIS. Thorough and long-term data collection will be needed to investigate whether the existing model can be applied to smaller manifestations or rather a specific model for these manifestations should be developed.

From our systematic review [10] we learned that external validation of existing prediction models of PPR and TTHR at MGs yielded poor results. Therefore, we doubt that the current model will be widely applicable, and we advocate the development of context-specific prediction models. However, we believe that the methods used and lessons learned from our study can be valuable for other researchers.

## Conclusions

We developed a prediction model for PPR and TTHR at MGs in Belgium. The resulting regression trees are easy to interpret and implement, and have good predictive performance for PPR at most manifestations the model was built upon. MG category, number of days, age class, and ambient temperature (for outdoor events in summer) were the most important predictor variables.

Validation with an external dataset revealed poor generalizability. More specifically, the MG categories “outdoor music” and “sports event” warrant further splitting in subcategories, and variables such as attendance, temperature and deployment of resources need to be systematically recorded in the future for superior prediction of MUR, and hence, optimal use of resources to provide medical care at MGs.

## Supplementary Information

**Additional file 1.**

**Additional file 2.**

**Additional file 3.**

**Additional file 4.**

**Additional file 5.**

**Additional file 6.**

## Data Availability

The datasets used and analysed during the current study are available from the corresponding author on reasonable request.

## Abbreviations

b_Tmax_: Slope of the linear regression of PPR_Tmax_ on T_max_
BRC: Belgian Red Cross
CART: Classification and regression trees
CF: City festival
EDM: Electronic dance music
EMS: Emergency medical service
EPV: Events per variable
ID: Indoor dance
IE: Indoor EDM
MedTRIS: Medical Triage and Registration Informatics System
MG: Mass gathering
MUR: Medical usage rate
OE: Outdoor EDM
OM: Outdoor music
PEF: Patient encounter form
PPR: Patient presentation rate
PPR_RTi_: PPR estimated by the regression tree for event i
PPR_RTRW_: PPR estimated by the regression tree for Rock Werchter
PPR_Tav_.: PPR adjusted for T_av_
PPR_Tmax_: PPR adjusted for T_max_
*R*^2^: Square of the correlation coefficient between observed and predicted values
RMI: Royal Meteorological Institute of Belgium
SE: Sports event
T_av_: Daily 24 h average temperature
T_max_: Daily maximal temperature
T_maxRW_: T_max_ for which PPR_Tmax_ = PPR_RTRW_
TRIPOD: Transparent Reporting of a multivariable prediction model for Individual Prognosis Or Diagnosis
TTHR: Transfer to hospital rate
VI: Variable importance
WHO: World Health Organization
