# Supplementary material for: Predicting medical usage rate at mass gathering events in Belgium: development and validation of a nonlinear multivariable regression model
Source: BMC Public Health. 2022 Jan 25;22:173. doi: 10.1186/s12889-022-12580-8 (PMC8789208; doi:10.1186/s12889-022-12580-8)
Supplement: Supplementary file 1 — Additional file 1. [file 12889_2022_12580_MOESM1_ESM.docx]

## Additional information overview

| **File name** | **File format** | **Title** | **Description** |
| --- | --- | --- | --- |
| Additional file 1 | .docx | Table A1 | TRIPOD Checklist for transparent reporting of development and validation of a multivariable prediction model |
| Additional file 2 | .docx | Table A2 | Characteristics of candidate predictor variables for the prediction models for PPR and TTHR |
| Additional file 3 | .docx | Supporting information A3 | Adjusting prediction model of PPR for temperature: methods and results |
| Additional file 4 | .docx | Table A4 | Characteristics of the 28 MGs included in the dataset for model development |
| Additional file 5 | .docx | Table A5 | Results of the univariable regression trees for each predictor variable |
