# Supplementary material for: Predicting medical usage rate at mass gathering events in Belgium: development and validation of a nonlinear multivariable regression model
Source: BMC Public Health. 2022 Jan 25;22:173. doi: 10.1186/s12889-022-12580-8 (PMC8789208; doi:10.1186/s12889-022-12580-8)
Supplement: Supplementary file 3 — Additional file 3. [file 12889_2022_12580_MOESM3_ESM.docx]

## Table A2. Characteristics of candidate predictor variables for the prediction models for PPR and TTHR

| **Variable name** | **Type of variable (original)** | **Source** | **Transformation** | **Remarks** |
| --- | --- | --- | --- | --- |
| **PPR and TTHR model** | | | | |
| **MG category** | categorical (6): city festival, indoor dance, outdoor music, indoor electronic dance music (EDM), outdoor EDM, sports event | own systematic literature review [1] | none |  |
| **Age** | continuous, median value | PEF | 4 age classes: children (<16 y); young adults (16-30 y); middle adults (>30 y); mixed/family (broad range) | mixed/family: (Q1 <21 y AND IQR >20 y) OR (Q1 <18 y AND IQR >15 y) |
| **Time** | categorical (3): day, night, day + night | online press archives | none | 8 PM was used as the cut-off time between day and night |
| **Number of days** | numerical, from 1 to 10 | PEF; online press archives | 3 categories: 1; 2; 3+ | only official event days were counted |
| **Number of attendees** | continuous (approximated) | organizers and online press archives | 5 categories: 10,000-20,000; 20,000-30,000; 30,000-100,000; 100,000-1,000,000; >1,000,000 | see Methods section of main text for handling of missing values |
| **Camping** | dichotomous: yes/no | online press archives | none | only “yes” for official camping facilities provided by the organizer of the MG |
| **Alcohol** | categorical (3): none, limited, unlimited | own judgement | none | none: events with minor spectators only; limited: before and after the main event only, i.e. for active sports events; unlimited: available throughout the course of the event |
| **Indoor/outdoor** | dichotomous | online press archives | none | tents on festival sites were considered as outdoor |
| **Bounded/unbounded** | dichotomous | online press archives | none | bounded: accessible by well-established manned entrance(s) only; unbounded: freely accessible |
| **Temperature** | continuous | archives of MeteoBelgië [2] | none | not inserted in the regression tree as a variable, but added afterwards as an adjusting factor (see Methods section of main text) |
| **Humidity** | continuous | archives of MeteoBelgië [2] | none | based on preliminary analyses, not further used in model development |
| **TTHR model only** | | | | |
| **Distance to hospital** | continuous | online tool [3] | 3 categories: ≤ 5 km; 5-10 km; > 10 km | address obtained from organizers or online press archives |
| **Time to hospital** | continuous | online tool [3] | 3 categories: ≤ 10 min; 10-20 min; > 20 min | address obtained from organizers or online press archives |
| **PPR predicted** | categorical (8): see Figure 1 | PPR model | none | PPR predicted by the crude regression tree (i.e. unadjusted for temperature) |

1. Van Remoortel H, Scheers H, De Buck E, Haenen W, Vandekerckhove P. Prediction modelling studies for medical usage rates in mass gatherings: A systematic review. PloS one. 2020;15(6):e0234977. Epub 2020/06/24. doi: 10.1371/journal.pone.0234977. PubMed PMID: 32574190; PubMed Central PMCID: PMCPMC7310685.

2. MeteoBelgië [cited 2019]. Available from: https://www.meteobelgie.be/klimatologie/waarnemingen-en-analyses/het-vervolg.

3. Afstand tot ziekenhuis berekenen (Calculate distance to hospital) [updated 19/01/2019; cited 2019]. Available from: https://atotz.bluelight.one/.
