# Supplementary material for: Predicting medical usage rate at mass gathering events in Belgium: development and validation of a nonlinear multivariable regression model
Source: BMC Public Health. 2022 Jan 25;22:173. doi: 10.1186/s12889-022-12580-8 (PMC8789208; doi:10.1186/s12889-022-12580-8)
Supplement: Supplementary file 4 — Additional file 4. [file 12889_2022_12580_MOESM4_ESM.docx]

## Supporting Information A3. Adjusting prediction of PPR for temperature.

### Methods

The association between the maximal daily temperature (T_max_) and PPR was investigated by linear regression analysis with data from the outdoor music festival Rock Werchter (36 datapoints), resulting in the following regression equation:

${PPR}_{T_{max}}=a+ b_{T_{max}}*T_{max}$ (1)

with PPR_Tmax_ the PPR in function of T_max_, a the intercept and b_Tmax_ the slope. This was rewritten as

${PPR}_{T_{max}}={PPR}_{{RT}_{RW}}+ b_{T_{max}}*{(T}_{max}-T_{{max}_{RW}})$ (2)

with PPR_RTRW_ the PPR estimated by the regression tree for Rock Werchter and T_maxRW_ the T_max_ for which PPR_Tmax_ = PPR_RTRW_ by cancelling out the second term of the equation. Finally, the equation was further generalised for other manifestations than Rock Werchter:

${PPR}_{T_{max}}={PPR}_{{RT}_{i}}+ \frac{{PPR}_{{RT}_{i}}}{{PPR}_{{RT}_{RW}}}*b_{T_{max}}*{(T}_{max}-T_{{max}_{RW}})$ (3)

with PPR_RTi_ the PPR estimated by the regression tree for event i. The factor PPR_RTi_/PPR_RTRW_ adjusts the slope b_Tmax_ proportionally to event i’s predicted PPR_RTi_, compared to the PPR_RTRW_ predicted for Rock Werchter.

Analogously, PPR_Tav_ was estimated as follows:

${PPR}_{T_{av}}={PPR}_{{RT}_{i}}+ \frac{{PPR}_{{RT}_{i}}}{{PPR}_{{RT}_{RW}}}*b_{T_{av}}*{(T}_{av}-T_{{av}_{RW}})$ (4)

### Results

We found a strong linear association of both T_av_ and T_max_ with PPR at the Rock Werchter festival. Introducing a quadratic or cubic term in each analysis revealed clearly non-significant associations. For T_max_, the equivalent for equation (1) was

${PPR}_{T_{max}}=-12.8+ 7.6*T_{max}$ (5)

with N = 36; p<0.0001; R² = 0.53. The regression tree predicted a PPR of 183/10,000 for Rock Werchter (terminal node 5 in Fig 1), corresponding with a T_max_ of 25.6°C in equation (5). Hence, it was rewritten as

${PPR}_{T_{max}}=183+ 7.6*{(T}_{max}-25.6)$ (6)

for predicted PPR at Rock Werchter, or as

${PPR}_{T_{max}}={PPR}_{{RT}_{i}}+ \frac{{PPR}_{{RT}_{i}}}{183}*7.6*(T_{max}-25.6)$ (7)

for any MG i that has a PPR predicted by the regression tree (PPR_RTi_). This way, the predicted PPR was adjusted with a term proportional to itself and to the difference between T_max_ and 25.6°C .

Analogously, for T_av_, the regression equation was

${PPR}_{T_{av}}=-34.2+ 10.6*T_{av}$ (8)

for Rock Werchter, and

${PPR}_{T_{av}}={PPR}_{{RT}_{i}}+ \frac{{PPR}_{{RT}_{i}}}{183}*10.6*(T_{av}-20.4)$ (9)

for any MG i.
