# Supplementary material for: Predicting medical usage rate at mass gathering events in Belgium: development and validation of a nonlinear multivariable regression model
Source: BMC Public Health. 2022 Jan 25;22:173. doi: 10.1186/s12889-022-12580-8 (PMC8789208; doi:10.1186/s12889-022-12580-8)
Supplement: Supplementary file 5 — Additional file 5. [file 12889_2022_12580_MOESM5_ESM.docx]

## Table A4. Characteristics of the 28 MGs included in the dataset for model development

| **Name** | **MG category** | **Nr. of edi­tions (2009-2016)** | **Atten­dance class^a^** | **Age class^b^** | **Alco­hol class** | **Indoor/ outdoor** | **Bounded/ unbounded** | **Time** | **Num­ber of days^c^** | **Cam­ping** | **PPR range (N/ 10,000)** | **TTHR range (N/ 10,000)** |
| --- | --- | --- | --- | --- | --- | --- | --- | --- | --- | --- | --- | --- |
| Afro-Latino | outdoor music | 5 | 2 | 2 | 2 | outdoor | bounded | day + night | 3 | yes | 90.9-110.5 | 1.1-3.2 |
| Antilliaanse Feesten | outdoor music | 8 | 3 | 3 | 2 | outdoor | bounded | night | 2 | yes | 52.7-117.1 | 0.8-2.1 |
| Antwerp Marathon & 10 Miles | sports event | 6 | 2, 3 | 3, 4 | 1 | outdoor | unbounded | day | 1 | no | 36.1-73.8 | 0.4-2.2 |
| Bassleader^d^ | indoor EDM | 5 | 1 | 2 | 2 | indoor | bounded | night | 1 | no | 83.1-121.3 | 2.5-4.4 |
| Blues Peer | outdoor music | 6 | 2 | 3 | 2 | outdoor | bounded | day + night | 3 | yes | 40.0-77.5 | 0.0-1.0 |
| Carnaval Aalst | city festival | 8 | 4 | 2 | 2 | outdoor | unbounded | day + night | 2, 3 | no | 4.0-21.9 | 0.3-3.8 |
| DayDream^d^ | outdoor EDM | 6 | 1, 2, 3 | 2 | 2 | outdoor | bounded | night | 1, 2 | no, yes | 100.3-212.7 | 1.0-7.5 |
| De Foute Party | indoor dance | 5 | 1 | 2 | 2 | indoor | bounded | night | 1 | no | 34.4-51.4 | 1.2-3.2 |
| Dranouter | outdoor music | 8 | 2, 3 | 2 | 2 | outdoor | bounded | day + night | 4 | yes | 167.7-626.2 | 1.4-6.4 |
| Gentse Feesten | city festival | 8 | 5 | 2 | 2 | outdoor | unbounded | day + night | 10 | no | 5.5-11.0 | 0.5-0.8 |
| Gordel­festival | sports event | 7 | 1, 2, 3 | 3, 4 | 1 | outdoor | unbounded | day | 1 | no | 21.2-67.2 | 0.2-2.4 |
| Graspop | outdoor music | 8 | 4 | 2 | 2 | outdoor | bounded | day + night | 3 | yes | 157.7-285.7 | 1.9-3.8 |
| I Love Techno^d^ | indoor EDM | 6 | 3 | 2 | 2 | indoor | bounded | night | 1 | no | 86.5-135.4 | 9.7-12.3 |
| I Love the 90s | indoor dance | 7 | 1 | 2 | 2 | indoor | bounded | night | 1 | no | 46.0-81.6 | 0.0-3.4 |
| Ieperfest | outdoor music | 5 | 1 | 2 | 2 | outdoor | bounded | night | 3 | yes | 217.0-416.2 | 3.0-10.0 |
| Laundry Day^d^ | outdoor EDM | 8 | 3 | 2 | 2 | outdoor | bounded | day + night | 1 | no | 73.7-178.5 | 0.7-2.3 |
| Maanrock | city festival | 7 | 4 | 2, 4 | 2 | outdoor | bounded | day + night | 2, 3 | no | 3.9-13.3 | 0.1-0.5 |
| Mano Mundo^d^ | outdoor music | 5 | 3 | 2, 4 | 2 | outdoor | bounded | day + night | 2 | yes | 17.1-45.7 | 0.3-1.2 |
| Marktrock^d^ | city festival | 6 | 4 | 2 | 2 | outdoor | bounded | day + night | 2, 3 | no | 5.8-13.1 | 0.0-0.5 |
| Pennen­zakkenrock | outdoor music | 8 | 1, 2 | 1 | 0 | outdoor | bounded | day | 1 | no | 79.4-255.7 | 0.0-1.0 |
| Reggae Geel | outdoor music | 8 | 3 | 2 | 2 | outdoor | bounded | day + night | 2 | yes | 82.4-193.3 | 1.0-5.9 |
| Reverze | indoor EDM | 8 | 1 | 2 | 2 | indoor | bounded | night | 1 | no | 75.0-113.0 | 1.5-5.0 |
| Rock Werchter | outdoor music | 8 | 4 | 2 | 2 | outdoor | bounded | day + night | 4 | yes | 127.9-223.2 | 1.1-2.9 |
| Ronde van Vlaanderen | sports event | 7 | 1 | 3 | 1 | outdoor | unbounded | day | 1 | no | 46.7-139.4 | 0.5-5.0 |
| Sfinks | outdoor music | 8 | 2, 3 | 4 | 2 | outdoor | bounded | day + night | 3, 4 | yes | 41.8-172.3 | 0.5-2.3 |
| Suikerrock | city festival | 8 | 4 | 2, 4 | 2 | outdoor | bounded | day + night | 3, 4, 5 | no | 12.4-23.5 | 0.1-0.7 |
| Summer­festival^d^ | outdoor EDM | 7 | 2, 3 | 2 | 2 | outdoor | bounded | day + night | 1, 2 | no, yes | 95.4-266.7 | 0.8-4.7 |
| TW Classic | outdoor music | 8 | 2, 3 | 3 | 2 | outdoor | bounded | day+ night | 1 | no | 58.0-135.6 | 0.6-1.8 |

^a^Attendance classes: 1: 10,000-19,999; 2: 20,000-29,999; 3: 30,000-99,999; 4: 100,000-999,999; 5: ≥1,000,000; cumulative for multi-day events

^b^Age classes: 1: children (<16 y); 2: young adults (16-30 y); 3: middle adults (>30 y); 4: mixed/family (( Q1 <21 y AND IQR >20 y) OR (Q1 <18 y AND IQR >15 y))

^c^Official event days only

^†^Event has been retired or moved abroad (as of July 2019)
