# Supplementary material for: Predicting medical usage rate at mass gathering events in Belgium: development and validation of a nonlinear multivariable regression model
Source: BMC Public Health. 2022 Jan 25;22:173. doi: 10.1186/s12889-022-12580-8 (PMC8789208; doi:10.1186/s12889-022-12580-8)
Supplement: Supplementary file 6 — Additional file 6. [file 12889_2022_12580_MOESM6_ESM.docx]

## Table A5. Results of the univariable regression trees for each predictor variable

| **Predictor variable** | **PPR (N = 194 MGs)** | | | **TTHR (N = 194 MGs)** | | |
| --- | --- | --- | --- | --- | --- | --- |
|  | **R²** | **nr. of splits** | **pattern**^a^ | **R²** | **nr. of splits** | **pattern**^a^ |
| **MG category** | 0.36 | 3 | CF 12; (ID, SE) 55; IE 98; (OE, OM) 148^b^ | 0.34 | 2 | CF 0.6; (ID, OE, OM, SE) 2.0; IE 5.9^b^ |
| **Attendance class** | 0.11 | 2 | >1,000,000 9; (30,000-1,000,000) 84; (<30,000) 134 | 0.07 | 2 | (>1,000,000) 1.2; (30,000-1,000,000) 2.3; (<30,000) 2.9 |
| **Age class** | 0.10 | 2 | (middle adults, mixed) 62; young adults 118; children 172 | 0.12 | 1 | (children, middle adults, mixed) 1.1; young adults 2.7 |
| **Timing** | 0 | 0 | no splits were created | 0.23 | 1 | (day, day + night) 1.5; night 4.0 |
| **Nr. of days** | 0.03 | 1 | (1 day, 2 days) 90; 3 or more days 122 | 0.05 | 1 | (2 days, 3 or more days) 1.7; 1 day 2.7 |
| **Indoor vs outdoor** | 0.01 | 1 | indoor 81; outdoor 106 | 0.20 | 1 | outdoor 1.7; indoor 4.4 |
| **Bounded vs unbounded** | 0.12 | 1 | unbounded 35; bounded 118 | 0.02 | 1 | unbounded 1.4; bounded 2.3 |
| **Camping** | 0.22 | 1 | no camping 67; camping 155 | 0 | 0 | no splits were created |
| **Availability of alcohol** | 0.05 | 2 | limited 55; unlimited 105; none 172 | 0.02 | 1 | none 0.6; (limited, unlimited) 2.2 |
| **Distance to nearest hospital** | n/a | n/a | n/a | 0 | 0 | no splits were created |
| **Time to nearest hospital** | n/a | n/a | n/a | 0.06 | 1 | 10-20 min 1.5; ((≤10 min, >20 min) 2.7 |
| **PPR predicted^c^** | n/a | n/a | n/a | 0.32 | 2 | (tn1, tn3, tn4) 0.8; (tn2, tn5, tn8) 2.2; (tn6, tn7) 4.2^d^ |

n/a: not applicable

^a^Pattern shows predicted PPR or TTHR per 10,000 visitors. Variable categories grouped between brackets belong to the same terminal node. The number of terminal nodes equals the number of splits + 1.

^b^CF: city festival; IE: indoor EDM; ID: indoor dance; OE: outdoor EDM; OM: outdoor music; SE: sports event.

^c^PPR as predicted with the model shown in Fig 1 (i.e. before adjustment with temperature).

^d^tn: terminal node, numbers of terminal nodes for PPR prediction correspond to those presented in Fig 1.
